# The ethical challenges facing the widespread adoption of digital healthcare technology

**DOI:** 10.1007/s12553-021-00596-w

**Published:** 2021-10-29

**Authors:** Azmaeen Zarif

**Affiliations:** 1 University of Cambridge School of Clinical Medicine, Cambridge, UK; 2 Gonville & Caius College, University of Cambridge, Trinity St, Cambridge CB2 1TA, United Kingdom

**Keywords:** Digital healthcare, Ethics, Prioritarianism, Rawls, Technology, Patients

## Abstract

With the rise of telemedicine, wearable healthcare, and the greater leverage of ‘big data’ for precision medicine, various challenges present themselves to organisations, physicians, and patients. Beyond the practical, financial, and clinical considerations, we must not ignore the ethical imperative for fair and just applications to improve the field of healthcare for all. Given the increasing personalisation of medicine and the role technology will play at the interface of healthcare delivery, a thorough understanding of the challenges presented is critical for future physicians who will navigate a novel environment. This article aims to explore the ethical challenges that the adoption of digital healthcare technology presents, contextualised at multiple levels. Potential solutions are suggested to initiate a discussion about the future of medicine and digital healthcare.

## 1 Introduction

Digital healthcare may be defined as the use of advanced technologies including ‘big data’, artificial intelligence and genomics to achieve health objectives for patients. The key argument for its widespread adoption, qualitatively interpreted as the continued and consistent presence across healthcare systems for a patient, stems not from an arbitrary normative assumption of the need for digitalisation but rather the several benefits it presents across various types of healthcare systems. This includes, but is not limited to, improving access to healthcare services, enabling greater autonomous active patient participation, facilitating better clinical decision making as well as allowing for cost savings and greater efficiency in the delivery of health services and, more broadly, better clinical outcomes [1–4]. The underlying technology of digital healthcare, however, necessitates a discussion into the philosophy underpinning the very nature of technology itself. Whilst some may argue that technologies enable the testing of hypotheses such that findings can be applied to pursue worthy goals, others counter that technologies make things appear which would not have existed without it (e.g. DNA or hypertension) [5, 6]. Svenaeus suggests that technology’s influence in shaping our experiences can change the very views we possess concerning the goals we pursue [7]. It is this concept of displacement that is important to consider within Western medicine’s patient-centred framework to ensure that human value is not disregarded in favour of the routinisation of novel digital health devices and procedures.

This tension between the benefits of technology in healthcare and the risks posed presents unique ethical challenges for widespread adoption. Fritz and Cox succinctly summarise the fundamental issue: whilst organisations balance financial as well as clinical obligations and physicians manage interpatient demands for current and future generations, they all do so in the absence of a philosophical framework to face novel healthcare challenges fairly and justly for the different users of healthcare services [8]. To identify the ethical challenges facing adoption, it is necessary to explore the multitude that are currently faced, and to categorise them contextually at multiple levels. We will analyse the challenges to the main stakeholders of the healthcare process as defined by the WHO classification of main users of digital healthcare, i.e. the patient, physician/healthcare providers and organisations [9]. Many authors have identified the principles of Rawls as a foundational approach to enable justice for different healthcare users. Whilst it is beyond the scope of this essay to cover the voluminous literature on the application of Rawls’ work to healthcare, before undertaking our discussion it is important to briefly summarise the key tenets of the Two Principles of Justice, as developed in *A Theory of Justice* [10]:

1. The Principle of Greatest Equal Liberty states that all citizens have an equal right to political liberty, freedom of speech and assembly, liberty of conscience, freedom of the person and expression, freedom of thought, and the right to hold personal property;
2. The Principle of Equality is further sub-divided into:
  - The Difference Principle, which states that socioeconomic inequalities can be just if they are arranged such that the least advantaged members of society receive the greatest benefit from them;
  - The Equal Opportunity Principle, where the arrangement of socioeconomic inequalities is such that they are attached to positions from which no individual is blocked from occupying, regardless of protected class characteristics.

Whilst Rawls did not include the right to health in his theory, academic discourse subsequently has subsequently attempted, and advanced, its coverage to cover the concept of health [11–13]. In the instance where these principles are found to be in conflict with each other, they can be ordered lexically: 1, 2b, 2a. In other words, The Greatest Equal Liberty Principle must be satisfied before The Equal Opportunity Principle which, in turn, must be satisfied before The Difference Principle. A principle cannot be applied till those of higher order are fully met or deemed inapplicable. In tandem with Rawls’ principles, which we will use to analyse the problem of achieving a socially just distribution of resources, we further employ the remaining three principles of Beauchamp and Childress’s four-principles ethical framework (respect for patient autonomy, beneficence in actions to place patient interests first, non-maleficence to ‘do no harm’ and justice for equitable distribution of resources) within the context of the intersection of technology with decision making in medicine and its practice, to stimulate debate regarding some of the ethical challenges faced [14]. A critical appreciation of said challenges can enable potential effective solutions.

## 2 Challenges for healthcare organisations

The cost of technology represents one of the largest challenges to organisations with respect to installation and subsequent maintenance [15]. The inability to directly assess intangible patient and cost benefits makes it difficult to value, and subsequently justify, the capital expenditure on new technology. Remote monitoring may be seen as a compromise compared to widespread installations. Proponents argue that reduced unnecessary hospital utilisation can act to lower healthcare costs, which may be especially pertinent in chronic disease management [16]. From a utilitarian viewpoint, this is beneficial in maximising effective outcomes for as many patients as possible. However, the literature suggests that remote monitoring does not act to decrease the cost for the vast majority [16]. Most studies suggest minimal, if any, cost savings, and sometimes increased costs; however, positive clinical outcomes were reported for the management of chronic conditions [17–19]. As per Rawls’ Difference Principle, the literature is not supportive of cost reductions benefiting the majority of those who are most disadvantaged. It has been suggested that underserved populations may even see greater costs in remote monitoring [16, 18]. Thus, it is critical to prevent a false sense of organisational autonomy from arising which may, without prudent oversight, lead to a shift in the healthcare organisation’s role from a patient-centred objective to a profit-centred agenda [16].

The concept of ‘new’ technology raises the issue of interoperability. Due to the timescales involved, integration and cross-access of data and usage between different systems or even different versions may be difficult [20–22]. Arguably, this stands in direct violation of the ethical obligation of organisations, derived from their fiduciary relationship with patients, to ensure that the incorporation of legacy systems meet integration standards [23].

## 3 Challenges for healthcare providers

Ethical challenges for physicians are thought to arise due to the “imprinting” of the necessity to provide the best possible medical care during medical training, to the extent that this implies the utilisation of the newest and most technologically advanced care [24]. With the advent of new healthcare technologies, this may lead to inappropriately rapid routinisation, defined as the social process which leads to the meaning of a new biomedical technology changing as participants become habituated to its use [24].

The role of the jurisdiction is important when economies of scale are considered as part of expansion. In the USA and the EU, doctors are required to obtain full licensing in the state in which the patients reside [25]. In the handful of cases so far, it has been deemed that healthcare professionals are ‘travelling’ to the patient for the virtual delivery of healthcare, thus a licence for that state is required [25]. When framed as per Beauchamp and Childress’s. Four Principles framework, the moral obligation of ‘Justice’ to achieve equity in health resource allocation can arguably be translated into a legal duty to not deny patients access to the highest standard of care due to their geographic location, thereby introducing further complications [14, 26].

Despite healthcare technology presenting significant opportunities to improve healthcare outcomes, inappropriate uptake must be acted against. The technological imperative refers to the inevitability of new technology and its essential nature which denotes the need for acceptance for societal good. Within healthcare, the domination of technology arguably modulates the purpose of healthcare from preservation and restoration under responsible autonomy to death prevention such that the patient is both ‘(the) battlefield and (the) prize’ [27]. This introduces the moral dilemma of using the patient themselves as a means to meet the end of death prevention, thereby violating one of the fundamental guiding principles of medical ethics: Autonomy. To explore this further, we must refer to the derivation of morality itself. Referring to the imperative implies an exigent choice with little room for manoeuvring. This occurs to the extent that it becomes unthinkable for doctors to not perform the treatment [24]. Thus, the moral imperative is derived from a social process-mediated origin such that, whilst novel technology is not mandated to be used, the imperative brings about normality as a result of routinisation and is indicative of how the social milieu has influenced the interpretation of its efficacy [24].

## 4 Challenges for patients

The implications of the moral imperative spill over into challenges presented to patients themselves. The need to ensure appropriate usage of expensive equipment (at least during the early adoption phase) and the moral imperative to utilise the new technology stands as a direct challenge to the need to preserve patient autonomy, as derived from the application of personalism to healthcare systems [28]. Their manifestation is their unique value which includes immanent properties such as free will [28, 29]. Their causative potential stands to be realised via the participation of the person in decisions centring them [28]. Thus, within the patient-centred framework of modern medicine, the challenge of the moral imperative presents a direct violation.

On the other hand, the position of technology does not need to be restricted to a false dichotomy of either the value-neutrality dictum or the value-ladenness thesis. The admittance of technological value does not automatically imply our subjugation to the technological imperative and reduction in autonomy. Cassell [5] proposes that the value-ladenness of technology is derived from its intrinsic characteristics which correspond to the deficiencies of human nature. Thus, handling of the value-ladenness of technology necessitates governance of our values and control of ourselves, whilst the management of its ethical challenges require the management of our ethos [5].

Despite addressing the minutiae, it is critical to consider the fundamental issue of patient access to digital healthcare technology and services. From a utilitarian viewpoint with regards to if, for example, remote monitoring enables greater access; for the majority, we know that no such improvements in access have been reported [16, 17]. Similarly, the data into whether or not remote monitoring promotes greater access for the most disadvantaged provides a similar result in that the Rawlsian perspective is not fulfilled [16]. In the context of digital health technology provisions, one can consider the disadvantaged to be the underserved, such as remote and rural communities as well as specific groups within the population as per the socioeconomic divide. Remote communities often lack the necessary financial and technological infrastructure whilst the technology itself may be biased to those with adequate levels of technological literacy [30]. This is, of course, under the assumption that the relevant technology has been offered or delivered in the first place. Ethics by design, which imposes a moral obligation on developers to design with aforethought of ethical implications to prevent any discrimination of access, remains a critical consideration for the planning phase of digital healthcare technology [31].

## 5 Conclusions

In tackling the challenges faced, the prioritarian approach to distributive justice has been proposed as a potential framework. Prioritarianism focuses on prioritising ‘the worse off in the distribution of advantages’ [32]. In the current climate, global healthcare is dominated by utilitarian principles with the rational and adopted action being that which produces the greatest good (e.g. numbers vaccinated and cases averted) [33]. In achieving scale and distributing innovative technology, this can be in contradiction to reaching the most disadvantaged [33]. On the other hand, prioritarianism is ‘sensitive to… (how) advantages are distributed among individuals rather than just concerned with the overall level or sum of them’ [32]. Ensuring that the needs of the disadvantaged (those who are ‘seldom heard’) are addressed requires less a concern with scale and rather over-correction to ensure that the privileged early adopters who are easier to reach do not dominate the conversation [33]. The principles suggested by Winters et al. include identifying what constitutes disadvantage (such that the social value of digital healthcare can be maximised for the worst off), designing and implementing digital healthcare technologies so that it addresses the needs of the worst off through their participation, and incorporating reflections about ethical obligations as digital healthcare researchers and practitioners [33].

Multiple challenges exist in this attempt to ensure the widespread and effective adoption of digital healthcare across different stakeholders and regions of a country as well as internationally. Even when Laennec introduced the stethoscope, it was accepted very gradually [34]. As per Rogers’s seminal ‘Diffusion of Innovations’[35], we are present in the early adopter phase, with the need, and gradual collection, of data (much so as the Philosophy of Science intended it) moving us along the hypothetical curve [36]. One would be correct to question whether, given the multitude of challenges faced, digital healthcare truly enables effective medical outcomes for the good of the patient. Within a Kuhnian context, the integration of digital healthcare acts to advance knowledge and further the physician’s role as part of a paradigmatic shift, whilst the empowerment of the patient enhances the overall doctor-patient relationship such that widespread adoption of digital healthcare technology is a goal worth pursuing for the future of the field.
